# Central Dental Association of Northern New Jersey

**Published:** 1897-01

**Authors:** 


### CENTRAL DENTAL ASSOCIATION OF NORTHERN NEW JERSEY.

A regular meeting of the Association was held on Monday evening, September 21, 1896, at the parlors of Mr. S. Davis, 943 Broad Street, Newark, N. J., the President, Dr. Walter Woolsey, in the chair, the usual Association dinner preceding the meeting.

*The President.*—The next thing in order is the discussion of the evening. On account of the absence of the essayist, the following subjects will be brought up for discussion: What amalgam do you individually consider the best? To what extent is the washing of amalgam an important feature in the production of a good filling? Dr. Hané, of Jersey City, will open the discussion.

*Dr. Hané.*—Mr. President, I don't know much about amalgam in this way; I think the discussion ought to have been on the question, What amalgam do you think best at the present time? We have amalgams of different kinds.

There are some amalgams that I used heretofore, or am using now, that may be considered good. When I commenced with amalgam I used the standard alloy, paying six dollars an ounce for it. I have since that time used the alba alloy, made by the S. S. White Dental Manufacturing Company, and I have also employed an alloy that is made by Olof Johanson in New York, at a cost of one dollar and fifty cents an ounce; and I find that we can make good fillings with any of these amalgams if we take care to prepare them properly, and have the material thoroughly dry, and not rub it into the cavity, but drive it home by tapping, somewhat as you would gold. The exception to this is when the first pieces are placed in the cavity they may be rubbed into the undercuts. One of the principal requirements for the success of amalgam, in my experience, is that the fillings must be properly polished; and you cannot finish the fillings at the time of inserting them; you must have your patient come back in two or three days or weeks, or months afterwards, and then complete the work. In this way you will find that almost any of these amalgams will wear a long time. There will be a little shrinkage, but without rolling up,—I do not know what other expression to use. I think that the proper finishing up of fillings is one of the principal elements of success.

I do not know that washing of amalgam has much to do with its wearing qualities. When I was in the habit of washing amalgam I used rectified spirits of wine or pure alcohol, then thoroughly drying in a towel before squeezing out the surplus mercury. That simply removes any dirt that may have been deposited on the amalgam in the office, and the oxidation caused by access of air into the bottle or wherever it may be kept.

*Dr. Barlow.*—Mr. President, I have used several different amalgams in my practice; I have used Welch's alloy, which is very satisfactory to me, and also Park & Carhart's amalgam, which I consider very good. In working my amalgam I am very particular to use a good quantity of mercury, working up the alloy so as to get it of a velvety texture and have the metals well amalgamated. I then press out all the mercury possible to remove, so that when it is used it will powder up; then insert it in that condition, and pack well into the cavity and the under-cuts. Bibulous paper is then used after the style that was once explained by Dr. Bonwill a number of years ago at Asbury Park. I have been practising that method of packing amalgam since that time. After the filling is inserted I trim and burnish it well, finishing the filling at once, and placing a polish on it that will remain. I am using an amalgam now that Dr. Meeker, I think, mentioned; it comes from Philadelphia; a sample was left with me, but I did not think much of it at that time; but I fell short of amalgam one day, and I used that, and was very well pleased with it. It works very nicely, gives good sharp edges, and has very little shrinkage, and it can be finished up if manipulated right. Everything is in the manipulation. I never place amalgam into a cavity in a plastic state and then work it up. It is almost in a solid condition, and inserted with spatulas and burnishers, using bibulous paper.

*The President.*—I have washed amalgam, but not recently. I do not think there is any great advantage in it, unless you have had it in the office for a long time. If you get it fresh and use it within a reasonable time there is no great gain in washing.

*Dr. Watkins.*—Mr. President, I do not agree with a good many dentists as to the manner of using amalgam. In inserting an amalgam filling we should take the same care as we would in placing in a gold filling. I believe there is more in the working of an amalgam and finishing than there is really in the amalgam itself, although there is a very great difference in amalgams. Now, unlike Dr. Barlow and many others, including Dr. Kirk, I use as little mercury as I can and have the amalgam hold together. Dr. Kirk gave us a long paper before the First District Dental Society of New York, in which he told us that we should always mix our amalgam with plenty of mercury, and then press out the mercury; that the mercury when thus pressed carried with it a certain portion of the metal,—what it was he did not tell us,—the getting rid of which improved the material, and left it in better condition, just why, he could not explain, in the same way that Dr. Barlow cannot tell us what combination he has when he has pressed out the mercury. Now, I do not use pressure. I mix the amalgam as dry as I can mix it, and have it hold together. I believe that amalgam should be mixed so as to have as much of the metal and as little of the mercury as possible, and then work it in with bibulous paper compresses so as to make it perfectly solid in the cavity, driving the mercury out while inserting the filling rather than in the preparation for the filling. And I would fill the cavity much more than full, wiping the mercury away as it rises to the top or sides, so that after the filling is completed the cavity is considerably fuller than is necessary; then cutting the surface down so as to secure proper shape and contour. The filling material in the cavity is perfectly hard, dense, and firm, and the instruments will give it a fine polish. Fillings placed in that way will change color very little, less, I think, than if placed in by any other method. They have strong edges, there will be very little bulging, they polish nicely, and there will be very little change of color, provided you use the rubber-dam while packing the amalgam. I would not use a matrix. I do not believe in them. Many dentists use them, and many will say that I am behind the times in objecting to them. I have used the matrix, but I saw no benefit in it, and abandoned it years ago. Where there are two approximate fillings to be made, and the space is quite large, I believe that this should be completely filled; I mean a space of say the thickness of a twenty-five- or fifty-cent piece, solidly with amalgam, and then separate the fillings by running a ribbon-saw between them. In that way you have all parts of the fillings of exactly the same density, and that is a secret of success in inserting amalgam fillings. The filling, after trimming with instruments, may be finished and polished with a fine tape. A beautiful contour is secured, and the edges are perfectly solid, close, and dense.

In regard to different kinds of amalgam: A few years ago I experimented with a number of different kinds. I had in the lot four or five different amalgams, including several White amalgams known as such, and other three-dollar amalgams. I made the experiments in this way: I would take two or three molars with large cavities, perhaps on the grinding, approximate, and buccal surfaces, place the dam on all of those teeth at the same time, and then fill one cavity with one kind of amalgam, another cavity with another kind, and another cavity with another kind; perhaps the buccal surface of a wisdom-tooth with one kind, the grinding surface of the same tooth with another; then a buccal cavity in the second molar with the same material placed in the grinding surface of the third molar; and on the grinding surface of the second molar the same material as in the buccal surface of the third molar, in that way mixing them up, and manipulating all with the same care, trimming the edges fairly around so that there would be no crumbling of the edges, and no weak points, and keeping the cavities perfectly dry while filling. I then marked those fillings and watched them from time to time; and in every instance, when I came to look at those fillings, I found Lawrence's amalgam standing pre-eminent so far as edge-strength was concerned. In color there was very little difference, so little that no one but myself, unless familiar with the different filling-materials used in the same teeth in different cavities, could discover any variation in color. Lawrence's amalgam had a little more of the steel color than the others, but the difference was slight, and it did not turn brown or dark, as it has the reputation of doing. In my hands Lawrence's amalgam has proved to be the best amalgam that I have used.

*Dr. Adelberg.*—Mr. President, may I ask Dr. Watkins whether he gave the other amalgams the same chance that he gave Lawrence's? Did you place Lawrence's amalgam in the buccal surface of the wisdom-tooth and the other amalgams in the grinding surface of the wisdom-tooth, and *vice versa?*

*Dr. Watkins.*—I mixed them up in every way.

*Dr. Richards.*—Mr. President, Dr. Watkins spoke of the mercury coming to the surface. There is a way to get rid of that, and it may be worth knowing, although I do not pretend that it was original with me. I do not wash my amalgam, although I formerly did. I make use of fine cloth for expressing the mercury. I make little tablets, cut into small pieces, and pack them all around, hard, on top. For the purpose of removing the excess of mercury I take small pieces of silver, the size of a very large pin-head, pick them up with pliers and hold them on the surface for a short time, one after another, and in that way take up all the excess of mercury. I then leave the filling a few days, finishing it up later when the patient returns for that purpose. In this way you secure a very satisfactory filling.

*Dr. Adams.*—Mr. President, this subject of amalgam is one of great importance. There are many men in our profession who profess never to have used amalgam, but I am incredulous. It is impossible to believe there is a dentist who considers the welfare of his patients who does not use amalgam to a greater or less extent. I have used several different amalgams, and my experience has been that the amalgam that serves the purpose with the best results, whether it be Welch's golden platinum alloy or any other amalgam that will not discolor and disfigure the tooth, but preserve it in its integrity, is the amalgam to use. Several years since, I abandoned washing amalgam because I did not see that any special good resulted. I take pains to express all the mercury possible, through a piece of chamois with a pair of pliers, and then pack the amalgam as solidly as it is possible to do. I do not think it is necessary to use the mallet. A pretty good filling can be made in this way, and one that, in my experience, will last for a long while. There is one question I would like to ask Dr. Watkins. Possibly I misunderstood him in his remarks, but I believe he stated that he never uses a matrix. I understood him, furthermore, to say that, if there were two approximate cavities—say, a posterior and an anterior approximate cavity—in two contiguous teeth, with a space between them the thickness of a silver dollar, that that space should be filled in filling the cavities. Am I right, Dr. Watkins?

*Dr. Watkins.*—I will acknowledge that, although I said a twenty-five-cent piece.

*Dr. Adams.*—And when that space is filled, he then with a ribbon-saw divides those two fillings; and the question I now want to ask is how he provides for the removal of the excess of amalgam that will necessarily be around the necks of those teeth? I want to know how he is sure that he has removed all the excess of amalgam and that a perfectly free surface is left there? It seems to me that the amalgam reaching over the necks of the teeth beyond the cavities will make a beautiful place for the accumulation of trouble.

*Dr. Watkins.*—I will answer that question by stating that I use Dr. Watkins's amalgam-trimming instruments for that purpose, and they do the work beautifully. After I use the ribbon-saw and get a space sufficient to draw a tape through, I enter with these instruments and trim around the necks of the teeth. The rubber dam has protected the cavity; the cervical wall of the cavity can be seen perfectly. I then take a tape and work around the filling until I have it as beautifully shaped as any gold filling that could be put in.

*Dr. Adams.*—I thank you. That is what I listened for in your first remarks and did not get.

*Dr. Watkins.*—There is one thing that we should be very particular about, and that is the finishing of the filling, and especially an amalgam filling, the cervical walls of approximate cavities.

*Dr. Louis C. Leroy*, of New York.—The few remarks that I have to offer are quite in harmony with those of Dr. Watkins. I agree with him thoroughly, especially in what he says about more than filling the cavity with amalgam. That is perhaps more important than any other part of the operation of filling, aside from the finishing, because plastic fillings are very liable to be rubbed away.

When I read the announcement of this meeting, sent out by your society, I wondered to what the society was tending. The American Society of Dental Surgeons some years ago sent out to its members a notice reading something like this: "Any member of this Society who shall hereafter refuse to sign a certificate pledging himself not to use any amalgam, under any circumstances, in his dental practice shall be expelled from the Society." There were eleven gentlemen expelled from that society under this rule. That was the society which preceded the American Dental Association. Evidently there has been a change of opinion since then, although Dr. Watkins says he rarely uses amalgam.

*Dr. Watkins.*—Oh, no. I did say, when I first spoke, that I would admit that I used amalgam.

*Dr. Leroy.*—I am glad you have corrected me in that. Let me quote Dr. Flagg on this occasion, who says, "The possibilities of amalgam are greater than those of all other filling-materials combined." I think so, too. If amalgam is used properly, as it should be, its possibilities for tooth conservation are greater than those of any other material. But, as in all other operations that we are obliged to perform, we must use judgment in the use of amalgam, giving the preference to gold in every instance where gold can be successfully used. I always endeavor to do that, but there are so many cases in which amalgam may be properly employed, even in contouring the whole tooth, using a matrix in the operation, that it is indispensable, and we can get marvellous results with it. I have many cases of such contouring in my practice that have given most excellent results. As to which is the best amalgam, it is difficult to say. I am using standard alloy; have had it in my office ever since I began practice; and it gives me such universally good results, and seems to be so unvarying in its excellence, that I have given it the preference. I have used Lawrence's amalgam, and it also seems to give very good results. The original formula was given by Dr. Flagg. Mr. DuBois was a patient of Dr. Flagg's, and they in conjunction brought forth the standard alloy.

Dr. S. B. Palmer ascribes the failure of fillings to "the incompatibility of the filling-material with the bone structure." The service which amalgam gives in tooth conservation depends principally upon the judgment of the operator, as the conservation of life depends largely upon the judgment of the physician.

I have been glancing over an article on amalgam and learn that it was first described under the name of silver paste, introduced in 1826 by Mr. Javeau, of Paris, and thought it might be of some interest to you as a matter of history.

I purchase my amalgam generally in four- or five-ounce quantities, particularly for the purpose of aging it, because there is something in the age of amalgam which gives better results. Another purpose is to get it at the lowest price, as I think that a man doing business should take advantage of all the opportunies that present themselves.

My next step in the handling of amalgam is to spread it out in a glass dish and run a magnet through it. You will be surprised to see what you get from it in that way; whether it be Lawrence's or Eckfeld & DuBois's or any other amalgam, I think you almost invariably find particles of steel in it. I used to wonder why there were little pin-like holes in some of my fillings after they were finished, and it occurred to me that they were due to particles of steel which became oxidized and disintegrated in the filling. After that I used a magnet, getting out of the amalgam varying quantities of little particles of steel.

As to washing the amalgam before using, although Dr. Flagg condemns that practice as being "absolutely detrimental," I believe that in my hands washing is an essential feature. I have for years almost invariably practised it. It removes the oxides. The washing should be done with pure alcohol. Alcohol does not dissolve the oxide, but simply holds it in solution; which is proved by the fact that the alcohol becomes almost clear again after being allowed to stand for a short time.

I think a most essential feature in the filling of a tooth with amalgam is to have the cavity perfectly dry when filling. I certainly am of the opinion that more of the failures with amalgam fillings are due to neglect in the matter of drying the cavities than to anything else. Many dentists are inclined to say, "Oh, it is only an amalgam filling; anything is sufficient." I do not think so. In some cases we may take the chances in using a napkin instead of the rubber dam. If the tooth-structure itself is conducive to good results, you may fill with almost anything or in any way, but with young people, below thirty years of age, I do not think one can safely take chances of that kind. We must be more cautious in the sterilizing and more positive in the drying out of the cavities.

A most essential feature in preventing the amalgam from showing through tooth-structure, so as to discolor it, is the method of lining the cavities with oxyphosphate. It should be used plastic, and the amalgam worked into the phosphate while this is in the plastic state. In that way you get a more homogeneous and positive union of the cement with the tooth-structure, and it undoubtedly makes a more positive tooth-conserving filling than any other that I know of. Where the cavities are exceedingly large I almost invariably follow this method, covering the base of the cavity with gutta-percha.

*Dr. Osmun.*—I do not know, Mr. President, that I have much to add to the remarks that have been made. My method of using amalgam is somewhat different from those that have been described here to-night, and, as it is individual methods that have been called for, I will give you mine. After I have prepared the cavity, having it perfectly dry, and protected with either a napkin or a rubber dam, I put in the first pieces of amalgam very dry and hard, and work it down well into the undercuts with a burnisher, using bibulous paper, and making the amalgam dryer and dryer, harder and harder, until I have the cavity full. I must take exception to Dr. Watkins's recommendation about filling the cavity in excess. I think that is a mistake. To my mind there is danger of loosening the filling from its anchorage, in trimming it down, if there is an excess of amalgam. I prefer to fill with amalgam as I would with gold, burnishing it down as well as I can around the edges. I do not think I would like to follow the plan of Dr. Watkins; in my hands I doubt if it would be successful. I do not see anything in it to recommend, and I can see a good many things about it that I would not like to commend. There is danger of rocking the fillings.

The washing of amalgam was dropped by myself a long while ago. I did not see that anything was to be gained by it, and it seemed to me that it was detrimental. I do not know that I can explain why I thought so.

In regard to the question of which is the best amalgam, I do not think it makes very much difference which one you use so long as you use it properly,—I mean the first-class amalgams, made by the best manufacturers, such as White, Johnson & Lund, and De Sanno & Hussey. I think De Sanno & Hussey's has a little more of the desirable velvety feeling. It is very important to have the mass thoroughly amalgamated. It is a good plan, when thoroughly mixed and amalgamated, to start in and do it again. I tell my office-boy to mix it until he gets tired, and then I mix it till I get tired, and in the end it feels like velvet. It is difficult for an operator to describe how he performs an operation. He does it over and over again, and after awhile it becomes a sort of second nature to him, but it is difficult to put the whole operation into words so as to convey the idea to another mind.

As to the relative value of amalgam and gold as filling-materials,

I do not think that needs discussion. Every operator who gives the question a second thought knows, beyond peradventure, that amalgam is one of his sheet-anchors,—he must use it, but at the same time use it with discretion.

*Dr. Richards.*—Mr. President, I would like to ask Dr. Osmun what he means by the rocking of fillings when he spoke of separating two consolidated, approximate fillings. Would it not press them in tighter?

*Dr. Osmun.*—I do not know just how to explain that. Two approximate cavities are never exactly of the same size; you never have the margins of one exactly opposite the margins of the other. They are seldom of exactly the same depth and width, with exactly the same kind of buccal and lingual surfaces; and when the surplus amalgam between them is cut away with a ribbon-saw, or with a spatula, you are liable to chip away pieces in pulling the saw back and forth, and at the same time you are very apt to rock the fillings and loosen them. That would be my criticism of the method of filling the spaces between two approximate cavities. I never feel very well satisfied if I cannot see the margins from the beginning to the end of the operation. I do not like this filling down into a blind hole where you cannot see what you are doing, and that is exactly the case in the use of the matrix. My experience is that I have not had as good success with it as I have without it.

*Dr. Watkins.*—Mr. President, Dr. Osmun says he does not believe in filling cavities more than full and then trimming away the excess, for fear it would rock the fillings. To me that seems very amusing, almost ridiculous. After a filling has been packed in solid, as I propose to pack it, I do not think there is any such thing possible as rocking that filling. If the amalgam is pressed down thoroughly as you go along, getting a good anchorage and a solid filling and a hard surface, there is no such thing possible as rocking it.

Dr. Richards spoke of cutting away between the fillings; that, instead of rocking them, it would have a tendency to pack and press them in harder. That is not so either, because they are pressed in as hard as they can be; they are as solid as a rock, and movement is impossible.

There is another matter that I wanted to speak about to-night, if permitted,—a matter that will be of interest to all of you, perhaps. It is new to me, although it may not be new to all of you.

We are all bothered more or less at different times with pericementitis; occasionally we will come across a tooth that has been filled for ten or more years; perhaps the canals have not been thoroughly filled, although it has not given trouble for a long time, but all at once the patient appears with a swollen gum and more or less pain; in other cases we have just filled the tooth, and the patient goes home to return in a few days with an inflamed tooth. Those cases have troubled us all, and dental science has failed to give us anything that will overcome the difficulty and give the patient sure relief. Now, there is a preparation, one of the derivatives from coal-tar, known as ammanol,—many of you may know all about it,—which meets these cases beautifully. Unlike the other coal-tar preparations, ammanol does not depress the heart's action; it is practically harmless, it will destroy fever, and it will relieve pain. I have tried it in a good many cases lately. A lady came into my office about three weeks ago in the middle of the night with a tooth that had been filled some ten years ago. She was suffering intensely, the tooth was elongated, she could not close her mouth, and there was considerable swelling of the gums. I gave her forty grains of ammanol,—not at one time, however. I gave her ten grains at once, and told her to go home and take ten grains more in an hour, and five grains more each hour until relieved. She took ten grains when she arrived home, and she went to bed and slept and had no more pain. About forty hours afterwards she came to me again with the tooth uncomfortable, and I gave her then a prescription to get more ammanol. She did so, and took twenty-five grains of that, and that was the last of the pain; there was no more inflammation, and I have not had occasion to touch that tooth since, and do not expect to. I have had several cases of a similar character,—one the case of a lady who suffered terribly, and who was brought to me by her physician. Another dentist had opened the root-canals and had never put anything into them except a piece of cotton with some medicine into the main pulp-chamber, then closed it up with another portion of cotton dipped in sandarac varnish. In about a week an abscess formed, and she was suffering intensely when this physician brought her in. I had to brace her up with whiskey and spirits of ammonia to quiet her and prevent fainting while I removed the cotton. I treated the tooth, closed it up, gave her some ammanol, and sent her home, and that was the last of the trouble with that tooth. Only day before yesterday another lady came in with a tooth very painful and elongated, and in about the same condition as the one first described. I gave her thirty grains of ammanol. She went home, and after taking about twenty-five grains was completely cured. This drug seems to relieve the pain and dispel the inflammation, and it is sure relief for the patient. Then in case of fever, you can break up a fever with it in a very short time. And if you want a stimulant for yourself to brace you up so that you can do an extra heavy day's work, take fifteen or twenty grains of ammanol in five-grain doses during the day, and you will not realize that you are tired at all: you can work right along.

*Dr. Adams.*—Mr. President, I want to add my testimony to the value of ammanol. I have been using it since last February in some cases of neuralgia in five-grain doses, and it has proved very effectual. I have given it to patients who were in great pain, to be taken at home, and it has almost invariably put them to sleep and made them comfortable. I do not administer it in larger doses than five grains, at intervals of two hours. In these cases in my experience it has dissipated the pain, and the patient has been made comfortable and happy.

*Dr. Watkins.*—It is an almost harmless remedy; there is no danger in giving fifteen to twenty-five grains. In some cases I have started in with ten or fifteen grains, and without any bad effect.

*A voice.*—By whom is it prepared?

*Dr. Watkins.*—The Ammanol Chemical Company, 36 East Fourteenth Street, New York. There was another preparation that I wanted to speak of, something in the same line, that I am experimenting with, and which I do not think any of you have tried. It works beautifully. I think I will give it to you at the next meeting.

*Dr. Adams.*—Mr. President, before the subject of amalgam is passed I want to say a few words. I was very glad to see the subject announced for the meeting to-night. There is one thing that my experience and observation has taught me, and that is that there are many men in our profession who seem to think that because a filling is to be of amalgam, there is no particular necessity of doing as thorough and careful work as they would do if they were making a gold filling, and I think that is a great error. My experience has been that when you begin to bur out a small cavity in the grinding surface of a molar, and follow the fissures leading from it, you generally find a much larger cavity than you expected. Now, if that cavity were to be filled with amalgam in the centre of the tooth, and finished off without burring out those fissures which extend in a crucial form across the crown of the tooth, at the end of a few months, when the patient returns, it will be found that an excavator can easily be inserted around the edges of the filling. I

feel prompted to say this because my observation has led me, in the twenty years that I have been in practice, to see the necessity for using as much care in the preparation of a cavity for an amalgam filling as there is in the preparation of a cavity for a gold filling; and the man who thoroughly removes the tissue around such crown cavities, and prepares them as he would prepare them for a gold filling, will find at the end of one, two, or three years that he has a filling there that is just as good as it was the day he finished it.

*Dr. Adelberg.*—Mr. President, I think Dr. Adams's last words struck the key-note in the making of a perfect amalgam filling,—the finishing. When an amalgam filling leaves your hands you never feel that it is completed until you see it again; that it is not a perfect filling until you have inspected it and know that it is thoroughly finished. If you would pay more attention to the finishing of amalgam fillings after they leave your hands the first day, having the patient return for the purpose of finishing, I think that this deficient edge-strength, shrinkage, and all that sort of thing will be done away with.

*Dr. Barlow.*—Permit me a word, Mr. President, in reference to the finishing of amalgam fillings with tape. That is something that I very seldom do. I use a burnisher and bibulous paper. I cut the bibulous paper into strips a couple of inches in length and fold them three or four times, then work them down about the filling, drawing them across the surface of the filling so as to conform it to the tooth. The bibulous paper carries away all the surplus mercury from the amalgam that may have been deposited below the margins, and the whole makes a nice clean filling. I consider that tape on an amalgam filling is altogether too coarse a thing to use; it tears away the amalgam instead of carrying it to an edge.

*Dr. Watkins.*—Mr. President, in answer to Dr. Barlow, I would like to say that I have used bibulous paper for twenty years, whenever I could not get fine linen tape. I do not know whether Dr. Barlow has had experience in using fine tape or not; but if he could use such tape as Dr. Adams has used, and such as I use now, he would not want to use bibulous paper except on very rare occasions. If the tape is fine, as it should be, and properly handled, it will polish down an amalgam filling beautifully, leaving cleaner cut edges and a smoother surface than bibulous paper can possibly do. An objection to bibulous paper is that it is liable to double-up and get thick, and in that way dig into the filling if it is at all soft, such as I imagine Dr. Barlow had reference to, and if a little too much pressure be used, then it breaks; but with a fine tape you can manipulate it exactly as you please, and it leaves a smooth surface.

*Dr. Barlow.*—Dr. Watkins may be more proficient in the use of the tape than I am, and I may be more proficient in the use of bibulous paper than he is.

*Dr. Mitchell.*—Mr. President, there is one important point in making an amalgam filling on the grinding surface of a molar, that is, to get the lip a little low, so that the impact of mastication will not bear on it too heavily, because I have found that sometimes in such cases the delicate walls are broken away by the motion of mastication, and the fillings drop out. That is one point that I think has not been brought out. I make more amalgam fillings than any other kind, because that is what they want down in Bayonne, and I find I have to look out for the leverage very carefully.

*Dr. Meeker.*—Mr. President, Dr. Flagg's series of experiments with amalgam were probably the most extensive and the most scientific of any that have been made in this country; he had specimens from the best metallurgists; his experiments were conducted on strictly scientific principles, the fillings being made in steel dies, and he showed the difference in the shrinkage of apparently the same amalgams when subjected to exact conditions. Now, these experiments show that the question of the best amalgam filling resolves itself into this one thing,—manipulation. If we have imperfect amalgam fillings, it is due to poor manipulation, or to abnormal conditions of the mouth. I know that I have made some poor amalgam fillings in my life, and I occasionally see some of my amalgam fillings put in twenty years ago that are still good. I find most of those old fillings made of Blackwood's amalgam, which is about the same as Lawrence's; the formula of both is nearly the same. I have used the De Sanno & Hussey's amalgam for six or eight months, and, like Dr. Osmun, I find it a very fine and velvety material; it is exceedingly smooth and velvety when you work it, and it gives a good edge-strength, and unless there is some abnormal condition of the mouth it will keep its color well. One thing about De Sanno & Hussey's amalgam is that the manufacturers come around at stated times and take away all your scraps, refine them, and give you as the result a good amalgam at fifty cents an ounce. I had about thirteen ounces the last time; I have used quite a little of it, and I am watching it. They add some metal to it, probably silver; but in whatever way they treat it, it wears just as well, apparently, as more costly amalgam.

I have here the printed proceedings of the meeting of 1895 of the State Society; it is somewhat ancient history, but I wish the members of the State Society who are here to-night, and who would like to have copies of these proceedings, would take them now and save me the trouble of sending them by mail.

*Dr. Leroy.*—There is one bad practice that I have noticed some dentists indulge in in preparing amalgam,—that is, using a two-per-cent. solution of sulphuric acid. I have always failed to see the real virtue of that, except that it removes some of the oxide, which I presume the washing of amalgam mainly means. If there are any here who have used sulphuric acid in that way, and who can say anything in defence of it, I would like to hear it.

*Dr. Meeker.*—I have used sulphuric acid, and I have used bicarbonate of soda, and I have used alcohol; but I do not see that washing with them makes any difference in the fillings in regard to color. I think it resolves itself down to careful manipulation of the filling.

On motion, the subject was passed.

*Dr. Watkins.*—Mr. President, there is another subject in which we are all interested, that is the painless extraction of teeth by means of the injection of some preparation into the gum. I have been using to some extent for that purpose a preparation called "eucaine." Eucaine is somewhat similar to cocaine, only less poisonous; it is practically safe. You can inject a five- or ten-per-cent. solution into the gum each side of the root, only a short time is required for its effect, from three to five or eight minims is a sufficient quantity, and you can extract the tooth usually with absolutely no pain. It has worked better in my hands than any other preparation that I have tried. The other day I had a case to prepare for an upper and under set, nothing but roots to remove. I made two injections, first from one wisdom-tooth to the central, then extracted all the roots on that side; then injected from the other wisdom-tooth to the central and extracted on the other side. There was no pain except in the removal of each of the wisdom-teeth. Perhaps I did not introduce quite enough of the remedy, or did not leave it long enough at that point. As to the rest, the patient said it was absolutely without pain. I have used this eucaine in quite a number of cases, and it works nicely, seems to be harmless, and leaves no sore mouth afterwards.

*Dr. Richards.*—Mr. President, what has been said about eucaine

I know to be correct. I had my druggist mix some for me in distilled water. I used it this morning on a very peculiarly nervous patient, in extracting a left lower cuspid; it was a very long root; I used two syringefuls,—a small glass hypodermic syringe.

*Dr. Watkins.*—I only used two syringefuls in the whole upper jaw.

*Dr. Richards.*—I admit it was a rather large quantity; she never moved a muscle, and I removed the tooth without any pain whatever. I was surprised at the result. I asked her if she felt badly afterwards, and she said, "No."

*Dr. Watkins.*—Eucaine comes in the same form as hydrochlorate of cocaine, and requires to be prepared in distilled water, then boiled. You set it in a tin basin of water and boil it. Then you have it absolutely pure and free from germicides. Wipe the gum clean with a weak solution of carbolic acid and you will be introducing no poison, and will create no inflammation.

*Dr. Meeker.*—There is one thing about cocaine that I think you ought to know: that is it decomposes in forty-eight hours. You need to prepare it fresh when you use it; if it is more than forty-eight hours old, it will not give good results.

*Dr. Watkins.*—Mr. President, I would like to say in regard to eucaine that, unlike cocaine, it will keep indefinitely; it will keep until it is used up.

