# Correction

**Published:** 1897-03

**Authors:** William H. Trueman


### Domestic Correspondence.

### CORRECTION.

To the Editor:

Sir,—The J in the next to the last line, page 49, of the January number should have been T. Mons. Ore Taveau has been credited with introducing amalgam into dental practice in 1826. In the first edition of his work, "Hygiène de la Bouche," published at Paris, 1826, he refers to fusible metal for filling cavities of decay; I find, however, no mention of silver paste. In the fifth edition of the same, Paris, 1843, page 236, following his reference to fusible metal and a modification of it made by adding mercury, he speaks of a paste that he has used seven or eight years with considerable advantage, which he has named "pâte d'argent (silver paste), and states that it is made by mixing silver in a very fine powder with a sufficient quantity of mercury and thoroughly incorporating them. When and in which one of Mons. Taveau's intervening works the above first appears I will be pleased to know.

William H. Trueman.

